# The Human Milk Microbiota Produces Potential Therapeutic Biomolecules and Shapes the Intestinal Microbiota of Infants

**DOI:** 10.3390/ijms232214382

**Published:** 2022-11-19

**Authors:** Martina Banić, Katarina Butorac, Nina Čuljak, Andreja Leboš Pavunc, Jasna Novak, Barbara Bellich, Saša Kazazić, Snježana Kazazić, Paola Cescutti, Jagoda Šušković, Jurica Zucko, Blaženka Kos

**Affiliations:** 1 Laboratory for Antibiotic, Enzyme, Probiotic and Starter Culture Technologies, Faculty of Food Technology and Biotechnology, University of Zagreb, Pierottijeva 6, 10000 Zagreb, Croatia; 2 Department of Life Sciences, University of Trieste, Via Licio Giorgieri 1, Ed. C11, 34127 Trieste, Italy; 3 The Ruđer Bošković Institute, Laboratory for Mass Spectrometry, Bijenička 54, 10000 Zagreb, Croatia; 4 Laboratory for Bioinformatics, Faculty of Food Technology and Biotechnology, University of Zagreb, Pierottijeva 6, 10000 Zagreb, Croatia

**Keywords:** human milk microbiome, infant intestinal microbiome, S-layer proteins, plantaricins, exopolysaccharides

## Abstract

Human milk not only provides a perfect balance of nutrients to meet all the needs of the infant in the first months of life but also contains a variety of bacteria that play a key role in tailoring the neonatal faecal microbiome. Microbiome analysis of human milk and infant faeces from mother-breastfed infant pairs was performed by sequencing the V1–V3 region of the 16S rRNA gene using the Illumina MiSeq platform. According to the results, there is a connection in the composition of the microbiome in each mother-breastfed infant pair, supporting the hypothesis that the infant’s gut is colonised with bacteria from human milk. MiSeq sequencing also revealed high biodiversity of the human milk microbiome and the infant faecal microbiome, whose composition changes during lactation and infant development, respectively. A total of 28 genetically distinct strains were selected by hierarchical cluster analysis of RAPD-PCR (Random Amplified Polymorphic DNA-Polymerase Chain Reaction) electrophoresis profiles of 100 strains isolated from human milk and identified by 16S RNA sequencing. Since certain cellular molecules may support their use as probiotics, the next focus was to detect (S)-layer proteins, bacteriocins and exopolysaccharides (EPSs) that have potential as therapeutic biomolecules. SDS-PAGE (Sodium Dodecyl-Sulfate Polyacrylamide Gel Electrophoresis) coupled with LC-MS (liquid chromatography-mass spectrometry) analysis revealed that four *Levilactobacillus brevis* strains expressed S-layer proteins, which were identified for the first time in strains isolated from human milk. The potential biosynthesis of plantaricin was detected in six *Lactiplantibacillus plantarum* strains by PCR analysis and *in vitro* antibacterial studies. ^1^H NMR (Proton Nuclear Magnetic Resonance) analysis confirmed EPS production in only one strain, *Limosilactobacillus fermentum* MC1. The overall microbiome analysis suggests that human milk contributes to the establishment of the intestinal microbiota of infants. In addition, it is a promising source of novel *Lactobacillus* strains expressing specific functional biomolecules.

## 1. Introduction

Evidence-based recognition of the health-promoting effects of probiotics led to the development of probiogenomics, a novel discipline of functional genomics that focuses on the molecular mechanisms of probiotic activities [1]. In addition, recent research in this field has focused on the development of next-generation probiotics, which, according to the US Food and Drug Administration (FDA), fall within the scope of “live biotherapeutic products” (LBPs) intended for the prevention and therapeutic treatment of disease [2]. If we consider the three different strategies during their selection [3], next-generation probiotics fall under the scope of the probiotic definition. However, these strains differ because they do not belong to the traditional *Lactobacillus-Bifidobacterium* group and have emerged from a detailed comparative microbiota analysis, or they belong to *Lactobacillus* and *Bifidobacterium* genera but have been altered using bioengineering tools. The evaluation of next-generation probiotics is based on molecular analysis extending beyond health-promoting effects to define probiotic-host interactions by exploiting “omic” technologies and conducting in vitro and in vivo studies [3,4]. The molecular mechanisms responsible for these effects are multifactorial and can be triggered by a range of biomolecules, which may be products of microbial metabolism or even molecules exposed at the cell surface, such as *Lactobacillus* surface (S)-layer proteins. S-proteins cover the bacterial cell surface and form a layer that provides the cell with a selective advantage in highly competitive habitats by protecting it from osmotic and mechanical stress, changes in pH values, bacteriolytic enzymes and antimicrobial peptides, radiation and bacteriophages [5]. S-proteins are synthesised by only a few *Lactobacillus* strains but are extremely important for the expression of their probiotic properties since they are in direct contact with the microenvironment and can mediate surface recognition, autoaggregation and coaggregation, adhesion to mucin, epithelial cells, extracellular matrix proteins, etc. [6,7]. Recently, research on S-proteins has focused on the nanobiotechnological application of these proteins as antigen carriers, artificial viral envelopes in gene therapy, and carriers in antitumour and antiallergic immunotherapy [8]. Bacteriocins, EPS and bioactive peptides are also recognised as potential biomolecules with health-promoting effects [9,10,11,12]. Bacteriocins are antimicrobial peptides that provide probiotic cells with an advantage in the competitive polymicrobial environment. These molecules act as signalling molecules in the producer’s quorum sensing interactions with other bacterial cells and as ligands in the interaction with the receptors of immune system cells and host epithelial cells, thereby directly influencing the regulation of signalling pathways [13]. Recently, they have been proposed as interesting candidates for biotherapeutic applications, even as an alternative to antibiotics [14]. EPS have a protective role during exposure of probiotic cells to the intestinal environment, exert a bifidogenic effect among intestinal microbiota, but also contribute to probiotic-host interactions, which are evident through antimicrobial, immunomodulatory, antitumour and cholesterol-lowering activities [10,15,16]. The human milk microbiome is recognised as a rich source of novel probiotic strains [17,18]. The human milk microbiome, together with bacterial species transferred during pregnancy and birth, contributes to the establishment of the intestinal microbiota of newborns [18,19]. Therefore, this study investigated the influence of the human milk microbiota on the infant’s intestinal microbiota. The study also reports the identification of unique bacterial isolates sampled from the human milk of Croatian mothers with a focus on the characterisation of specific cellular molecules, particularly S-layer proteins, bacteriocins and EPSs, which may support their use as probiotics and thus have potential as therapeutic biomolecules.

## 2. Results

### 2.1. The Composition of Human Milk and Infant Faecal Microbiome 

**Figure 1:**
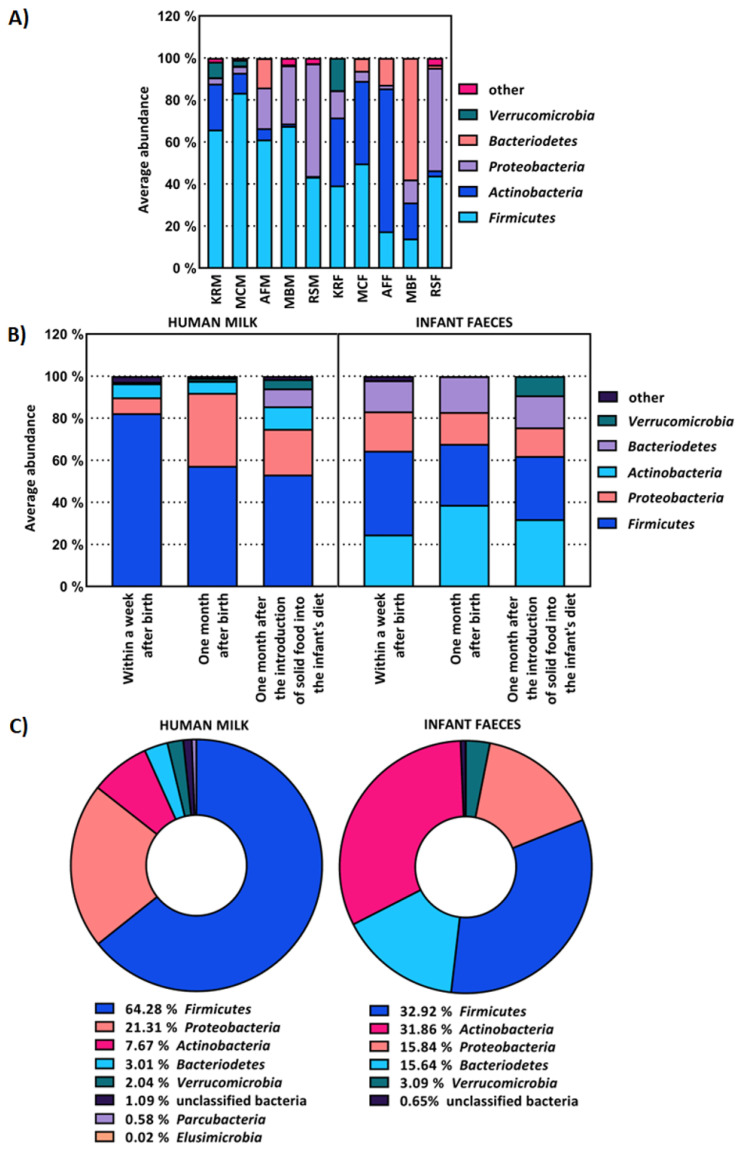
Average abundance of operational taxonomic units (OTUs) at phylum level of: (**A**) human milk samples from the same mother (M), i.e., faeces (F) from the same infant, (**B**) all human milk and infant faeces samples collected within one week and one month after birth, and one month after the introduction of solid food into the infant’s diet, (**C**) all human milk and infant faeces samples. OTUs with an average representation of less than 2% are classified as other.

MiSeq sequencing revealed that each sample of the human milk and infant faeces microbiome had a unique composition. The abundance of phyla in the human milk microbiome differed between mothers (Figure 1A left). The relative abundance of *Firmicutes* ranged from 43.36% to as high as 83.37%, *Actinobacteria* from 0.46% to 21.89%, *Bacteroidetes* from 0.17% to 13.94%, *Proteobacteria* from 2.86% to 53.46% and *Verrucomicrobia* from 0% to 7.38%. The faecal microbiome of infants was dominated by *Firmicutes* (32.92%), *Actinobacteria* (31.86%), *Proteobacteria* (15.84%), *Bacteroidetes* (15.64%) and *Veruccomicrobia* (3.09%), which correlated with the relative abundance of the same phyla in the human mother’s milk microbiome (Figure 1A). The relative abundance of phyla in the faeces differed between infants (Figure 1A right), as *Actinobacteria* ranged from 2.54% to as high as 67.98%, *Bacteroidetes* from 0.08% to 57.89%, *Firmicutes* from 14.05% to 49.75%, *Proteobacteria* from 1.79% to 48.74% and *Verrucomicrobia* from 0.00% to 15.35%. When comparing the composition of microbiome samples collected within one week, then one month after birth and one month after the introduction of solid food into the infant’s diet, it was found that the composition of the human milk microbiome changes during lactation, which also affects the composition of the infant’s faecal microbiome. The proportion of *Actinobacteria* and *Verrucomicrobia* increased over time in both human milk and the infant’s faeces, while the proportion of bacteria from the phylum *Bacteroidetes*, which degrade complex plant polysaccharides, increased in the infant’s faeces up to 34 times after the introduction of solid food into the infant’s diet (Figure 1B). The human milk microbiome showed a greater number of phyla compared to the infant faecal microbiome (Figure 1C), which was also confirmed by the literature data [20,21].

The human milk microbiota was dominated by bacteria from seven phyla, predominantly belonging to *Firmicutes* and *Proteobacteria*, whereas the faecal microbiomes from infants were found to contain bacteria from five phyla, predominantly *Firmicutes* and *Actinobacteria.*

**Figure 2:**
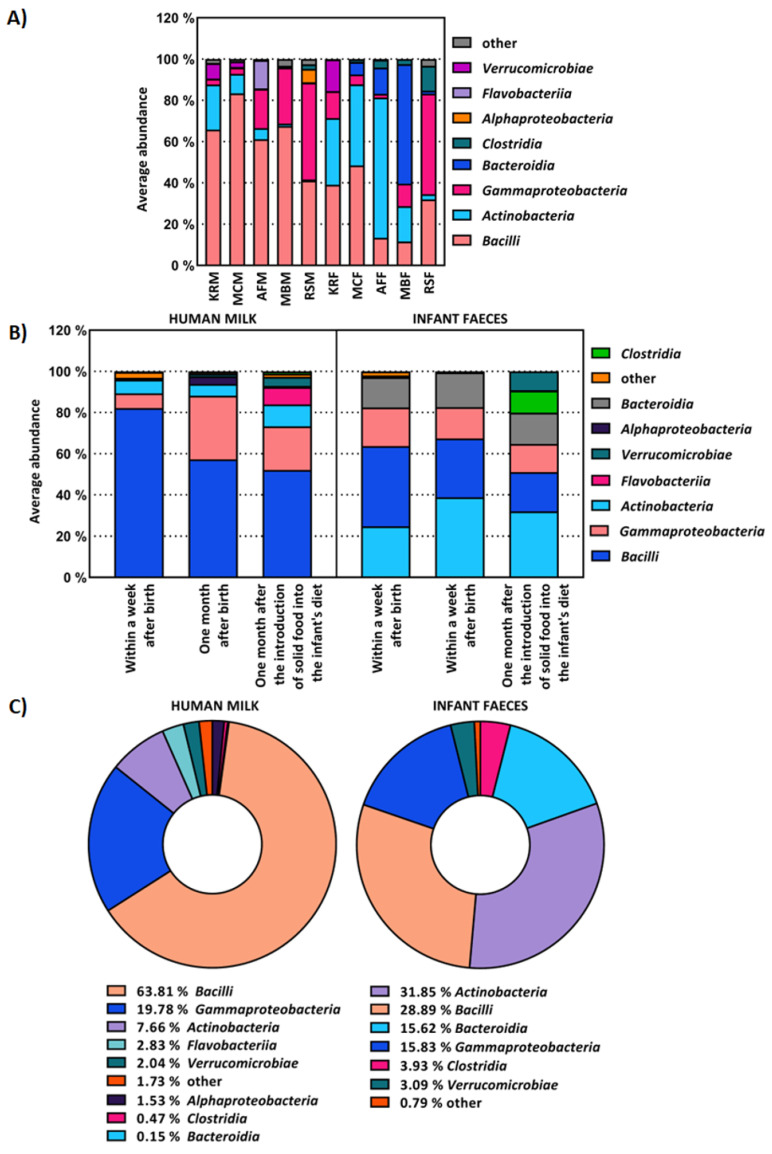
Average abundance of OTUs at class level of: (**A**) human milk samples from the same mother (M), i.e., faeces (F) from the same infant, (**B**) all human milk and infant faeces samples collected within one week and one month after birth, and one month after the introduction of solid food into the infant’s diet, (**C**) all human milk and infant faeces samples. OTUs with an average representation of less than 2% are classified as other.

The average abundance of bacterial OTUs at the class-level present in all human milk and faecal samples from the same mother and infant, respectively, was also examined (Figure 2A). The most abundant bacterial classes in the infant faecal samples were *Bacteroidia*, *Flavobacteria*, *Bacilli*, *Clostridia*, *Gammaproteobacteria* and *Verrucomicrobiae*, as in the milk samples of their mothers. A low abundance of bacteria of the classes *Actinobacteria*, *Bacteroidia*, *Flavobacteria* and *Verrucomicrobia* was detected in faecal samples from the infants, as well as in the human milk they were fed (Figure 2A). The results showing the average abundance at the class level in human milk and faeces samples collected within one week or month after birth and one month after the introduction of solid food into the infant’s diet are shown in Figure 2B. In human milk microbiome, the abundance of bacteria from the *Bacilli* class decreased from 82.12% to 57.21% during lactation and finally to 52.10% at the end of the experiment, i.e., 1 month after the introduction of solid food into the infant’s diet. A similar trend was observed in the faecal microbiome of infants, where the abundance of bacteria from the *Bacilli* class decreased with infant age from 38.99% to 28.60% and finally to 19.10%. The average abundance of *Clostridia* was low across all human milk samples, increasing slightly from 0.03% to 0.11% to 1.10% during lactation. The lower proportion of *Clostridia* in the faeces of infants was detected when the infants were exclusively breastfed. In the infant faeces samples collected within one week and one month after birth, their relative abundance was only 0.78% and 0.32%, respectively, while it increased to 10.67% after the introduction of solid food into the infant’s diet. The average composition of the human milk and infant faecal microbiome at the class level is shown in Figure 2C. The microbiota of human milk was dominated by *Bacilli* (63.81%), *Gammaproteobacteria* (19.78%), *Actinobacteria* (7.66%), *Flavobacteria* (2.83%), *Verrucomicrobiae* (2.04%), *Alphaproteobacteria* (1.53%), *Clostridia* (0.47%) and *Bacteroidia* (0.15%). In the faeces of infants, the most abundant bacterial classes were *Actinobacteria* (31.85%), *Bacilli* (28.89%), *Gammaproteobacteria* (15.83%), *Bacteroidia* (15.62%), *Clostridia* (3.93%), and *Verrucomicrobiae* (3.09%). 

**Figure 3:**
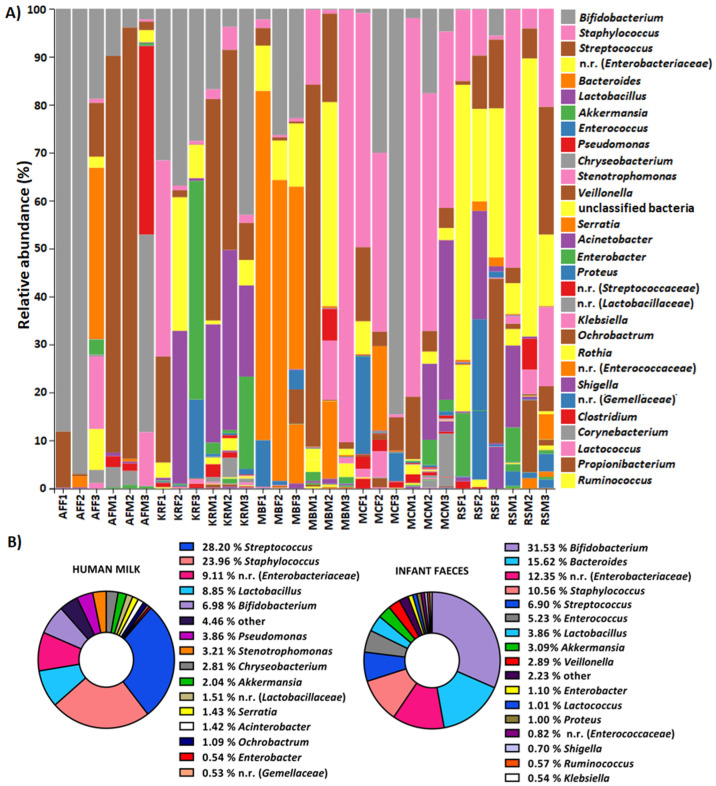
Relative abundance of OTUs at genus level of: (**A**) human milk samples from the same mother (M), i.e., faeces (F) from the same infant, (**B**) all human milk and infant faeces samples. OTUs with an average representation of less than 0.5% are classified as other. n.r.—not recognised genus from the family listed in parentheses.

Based on the analysis of relative composition at the genus level, the uniqueness of each sample is observed, as well as the connection of the composition of the microbiome of human milk with the microbiome of its brestfed infant (Figure 3A). The average abundance of *Bifidobacterium*, *Bacteroides*, *Staphylococcus*, *Enterococcus*, *Lactobacillus* and *Stenotrophomonas* was lowest in the faecal samples of infants fed with human milk, with the lowest proportion of these bacteria also observed. The highest average representation of bacteria of the genera *Bacteroides*, *Chryseobacterium*, *Staphylococcus*, *Lactobacillus*, *Clostridium*, *Enterobacter*, *Proteus*, *Serratia*, *Acinetobacter* and *Akkermansia* was also recorded in the human milk and infant faecal samples. The average abundance of bacteria of the genus *Bifidobacterium* increased during lactation and in the faecal samples, from 5.80% to 10.02% and from 24.64% to 31.88% at the end of the experiment, respectively. 

At the genus level, bacteria of the genera *Streptococcus* (28.20%) and *Staphylococcus* (23.96%) were found to predominate in human milk, but high proportions of *Lactobacillus* (8.85%) and *Bifidobacterium* (6.98%) were also detected. Bacteria of the genera *Bifidobacterium* (31.53%) and *Bacteroides* (15.62%) predominated in the faeces of the infants (Figure 3B). 

**Figure 4:**
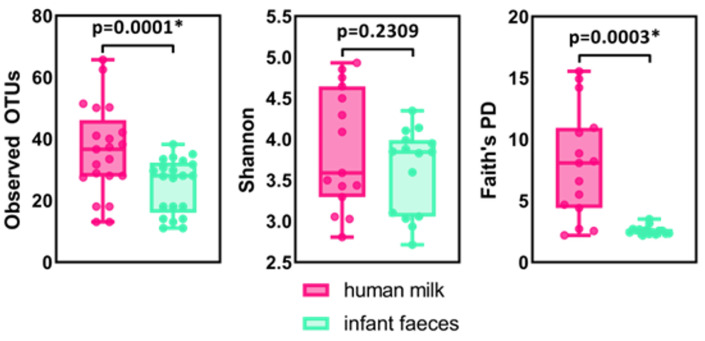
α-diversity boxplots summarising bacterial diversity of human milk and infant faecal samples using Observed OTUs, Faith’s phylogenetic diversity (PD) and Shannon indices. Asterisks (*) indicate a significant difference (*p* < 0.01) in bacterial diversity between human milk and infant faecal samples. Statistical analysis was conducted using Two-Way ANOVA.

The α-diversity of the human milk and infant faeces microbiome was also assessed using the “Observed OTUs”, “Faith’s Phylogenetic Diversity (PD)”, and “Shannon” metrics (Figure 4). The graphs of the dilution curves reached a plateau, indicating that the sequencing depth was sufficient and that a greater number of sequences per sample would not have provided more reliable data or resulted in a greater number of species (Supplementary Figure S1). The samples of human milk and infant faeces with the lowest α-diversity belonged to the same mother-breasted infant pair (Supplementary Figure S1). Human milk showed significantly higher (*p* = 0.0003) phylogenetic diversity than infant faeces using Faith’s PD metric (Figure 4). Significant (*p* = 0.0001) compositional differences between human milk and infant faecal samples were also observed at the OTU level when using the Observed OTUs metric. The higher α-diversity of the human milk microbiome implies that it is richer in species than the microbiome of infant faeces. To further predict microbial diversity, we used the Shannon index, which combines both bacterial richness and evenness and is more responsive to rare species rather than dominant species in terms of species richness. The analysis revealed that there was no significant difference (*p* = 0.2309) in the bacterial diversity of the human milk microbiome compared to the infant faecal microbiome. Interestingly, the human milk and infant faecal samples with the lowest α-diversity belonged to the same mother-breastfed infant pair (Supplementary Figure S1). 

**Figure 5:**
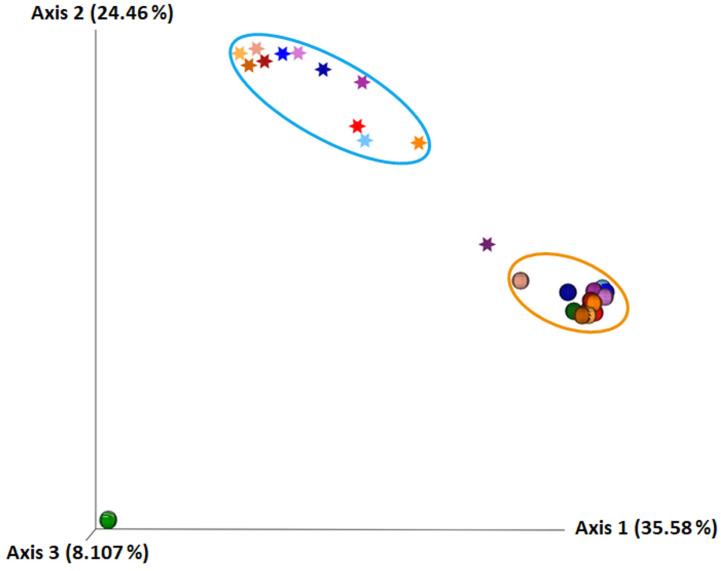
PCoA analysis of β-diversity calculated with the Unweighted UniFrac metric. Human milk samples are represented by an asterisk and faecal samples by a circle. Samples from the same pair of mother and breastfed infant are marked with the same colour, with the samples with the lightest shading of each colour collected within one week after birth, with the medium shading one month after birth, and with the darkest shading one month after the introduction of solid food into the infant diet. The blue ellipse surrounds a cluster of human milk samples, whereas the orange ellipse surrounds a cluster of infant faecal samples.

A qualitative Unweighted UniFrac metric was used to calculate β-diversity, in which samples that have a similar composition of evolutionarily close bacterial communities are characterised by a small UniFrac distance. The β-diversity was used to estimate the dissimilarity of microbiome composition. By using PCoA analysis, samples were placed into a three-dimensional frame based on the distance calculated using the unweighted UniFrac metric, forming two clusters, one comprising human milk samples and the other infant faecal samples (Figure 5). PCoA analysis showed that each sample was unique, but it also demonstrated greater similarity between the same source samples (human milk or infant faeces). The cluster representing the faecal samples is more compact, implying that the infant faecal microbiome is more similar in composition than the human milk microbiome.

### 2.2. Synthesis of Potential Biomolecules by LAB Strains Isolated from the Human Milk Microbiota

The results of human milk microbiome analysis revealed a relatively high proportion of beneficial *Lactobacillus* strains, prompting the selection of lactic acid bacteria (LAB) strains expressing specific cellular components that have potential as therapeutic biomolecules. A total of 100 bacterial strains were isolated from the collected human milk samples, i.e., 20 from each mother’s milk. In order to exclude genetically identical isolates, DNA fingerprinting of all isolated strains was performed using the RAPD-PCR method. Electrophoresis of the RAPD-PCR products yielded unique genetic profiles of each strain, which were then classified by hierarchical cluster analysis into 28 clusters distributed across five separate dendrograms (Supplementary Figure S2). Since bacteria with identical RAPD-PCR fingerprints isolated from the same source are considered to belong to the same strain, one representative of each cluster generated by the dendrogram was selected for further analysis. A total of 28 genetically distinct strains selected by dendrogram analysis were phenotypically characterised [22] and identified by sequencing of the 16S rRNA gene (Table 1). The bacterial isolates were identified as genera *Enterococcu*s (46%), *Staphylococcus* (27%) and *Lactobacillus* (22%), while 5% of the isolated bacteria belonged to the genus *Streptococcus*.

In order to detect S-layer proteins as potential biotherapeutic molecules, the surface proteins of human milk bacterial isolates were extracted, and their electrophoretic separation was performed. The strain *Lactobacillus helveticus* M92, which produces S-layer proteins, was used as a positive control, and the strain *Lactiplantibacillus plantarum* D13, which does not produce S-layer proteins, was used as a negative control. The protein profiles obtained by SDS-PAGE analysis revealed S-layer proteins in a range of 45-55 kDa solely in eight strains, MB1, MB2, MB4, MB9, MB12, MB13, MB19 and MB20 (Supplementary Figure S3). Hierarchical cluster analysis of the protein profiles classified these strains into four clusters (Table 1). The S-layer proteins were further analysed by SDS-PAGE coupled to LC-MS and identified by Mascot database search. The result showed that the S-layer proteins were homologous to the S-layer protein of *Levilactobacillus brevis* strain, accession WP_097547033.1 (Table 2). The MASCOT protein score of the analysed S-protein samples from *L. brevis* strains MB1, MB2, MB13 and MB20 was far above the cutoff values, implying higher reliability of the homology match. 

**Figure 6:**
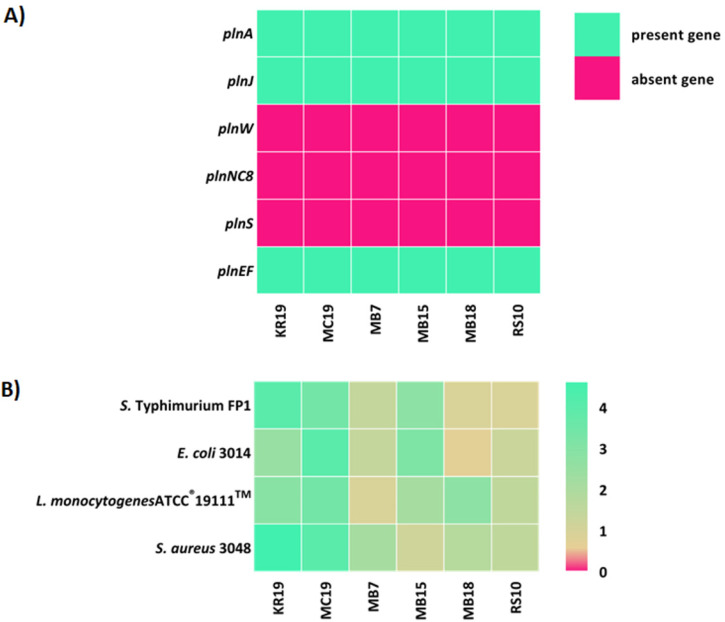
(**A**) Plantaricin-related genes of *L. plantarum* KR19, MC19, MB7, MB15, MB18 and RS10 strains detected by PCR with plantaricin structural gene-specific primers. (**B**) Heatmap comparison of antimicrobial activity of selected *L. plantarum* strains tested against potential pathogens using an agar spot-test, expressed as effective inhibition ratio (EIR). According to Coeuret et al. [23], EIR < 0.5 indicates weak inhibition; EIR > 1.5 strong inhibition, while values in between indicate medium inhibitory activity.

As a first step towards the detection of bacteriocin producers, the presence of genes for bacteriocin biosynthesis in the genomes of the examined LAB strains isolated from human milk was tested by PCR analysis with six different primer pairs (Supplementary Figure S4). Plantaricin-related genes were detected in six *Lactiplactibacillus plantarum* strains (Figure 6A). PCR analysis confirmed that *L. plantarum* strains KR19, MC19, MB7, MB15, MB18, and RS10 are potential producers of plantaricinsas they contain three genes (*plnA*, *plnEF* and *plnJ*) responsible for the production of plantaricins PlnA, PlnEF and the peptide PlnJ of plantaricin PlnJK (Supplementary Figure S4, Figure 6A). Therefore, their antagonistic activity was examined by agar spot-test method against *S. aureus* 3048, *L. monocytogenes* ATCC^®^ 19111™, *E. coli* 3014 and *S. enterica* serovar Typhimurium FP1 (Figure 6B). According to the results, *L. plantarum* KR19 and MC19 showed the strongest antimicrobial activity against the potential pathogens tested. The same strains also showed strong antibacterial activity against the related *Lactococcus* and *Enterococcus* strains [22].

**Figure 7:**
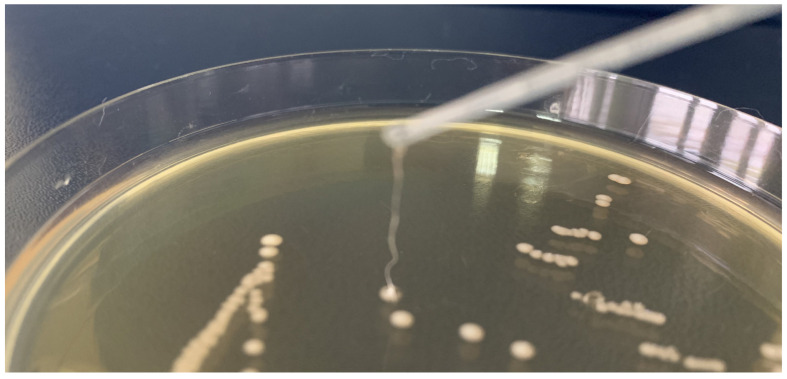
“Ropy” phenotype of *Limosilactobacillus fermentum* MC1 strain.

**Figure 8:**
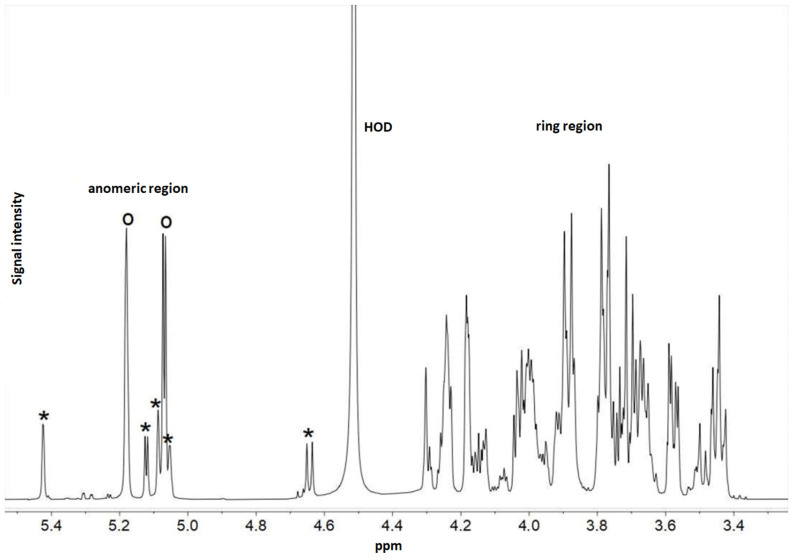
^1^H NMR spectrum of MC1 EPS-b recorded in D_2_O at 500 MHz at 50 °C. Protons of the ring and anomeric regions are shown. Open circles (o) denote the two most intense anomeric resonances belonging to the main polysaccharide, while asterisks (*) indicate the five less intense anomeric peaks, indicating another polymer present in minor amounts.

An EPS-producing LAB strain can be easily detected by observing the “ropy” phenotype. A positive indication of EPS production is the formation of long strands when the grown colony is touched. Among the LAB strains isolated from human milk, only one strain, *Limosilactobacillus fermentum* MC1, displayed this phenotype after the overnight growth on the MRS agar medium (Figure 7). The production of EPS bound to the cell surface (EPS-b) was further confirmed by recording the NMR spectra of purified and freeze-dried EPS (Figure 8). The ^1^H NMR spectrum showed signals typical of carbohydrates, with ring proton resonances of carbohydrates in the region 4.4–3.4 ppm and anomeric protons in the interval 5.5–4.6 ppm. In the anomeric region of the MC1 EPS-b ^1^H NMR spectrum (Figure 8), two main resonances were detected at 5.18 and 5.07 ppm with identical integration areas, suggesting the presence of a polysaccharide composed by a disaccharide repeating unit. Moreover, in the anomeric region of the MC1 EPS-b ^1^H NMR spectrum, five less intense peaks were detected at 5.42, 5.12, 5.09, 5.05 and 4.64 ppm, all having an integration area of about 20% with respect to the main peak at 5.18 ppm, indicating the presence of another polysaccharide probably constituted of five different sugars.

## 3. Discussion

The microbiota of human milk is unique and site-specific. Initially, the bacteria present in human milk were thought to be the result of contamination. However, this has been disproved by the isolation of bacteria from samples collected under aseptic conditions and by genotypic analysis, which showed that the bacteria present in human milk are genotypically different from those present on the skin [24]. Research has confirmed that human milk contains a unique microbiome that is more diverse than the gut microbiome [20] and that the bacteria present in breast milk, in addition to those acquired during pregnancy and at birth, are involved in the primary colonisation of infants’ gut [25]. In addition to being a rich reservoir of potential probiotic microorganisms, human milk also contains 200 structurally distinct indigestible oligosaccharides that act as prebiotic substrates, modulate the immune system, influence the proliferation and differentiation of intestinal epithelial cells and prevent the adhesion of pathogens [26]. Human milk has the greatest influence on the composition of the infant’s intestinal microbiota in the postpartum period [27]. By analysing the relative abundance of OTUs at the phylum level, it was found that the human milk microbiome of each mother, just like the intestinal microbiome of each infant, is unique. The relative abundance of each phylum in the faecal samples varied greatly between infants, which is consistent with Yatsunenko et al. [28], who have shown that the intestinal microbiome of a newborn has lower bacterial diversity compared to the intestinal microbiome of an adult, with much greater compositional variation between different individuals. According to the results, the influence of the composition of the human milk microbiome on the faecal microbiome of infants is noticeable, which is to be expected since an infant consuming about 800 mL of human milk daily ingests about 10^5^–10^7^ commensal bacteria [29]. Moreover, the results showed that mother-associated microbes were among the first gut colonisers, which was most apparent in the meconium samples. During lactation, in addition to the nutritional and immune composition, the composition of the microbiota of human milk also alters. Colostrum is the first milk produced, and after 2–5 days, secretion of transitional milk begins for about two weeks. Finally, 4–6 weeks after the birth of the infant, the milk is considered fully mature [30]. Wan et al. [31] showed that during lactation, the relative abundance of *Proteobacteria* increases while the relative abundance of *Firmicutes* decreases, which was also confirmed in this study. In addition to the composition of the microbiome of human milk, the microbiome of the infant’s faeces also changed with the child’s age. According to Pannaraj et al. [25], the proportion of *Proteobacteria* in faeces decreases with the infant’s age, which was also confirmed in this work. According to Pannaraj et al. [25], continued breastfeeding after the introduction of solid food into the infant’s diet reduces the proportion of butyrate-producing *Firmicutes* in the infant’s gut microbiome, often associated with an obese phenotype in mice and humans. The highest proportion of bacteria from the phyla *Proteobacteria* and *Firmicutes* in the first collected faecal samples can also be explained by the fact that they represent the dominant microbiota of the placenta and are the first colonisers of the infant’s intestines [32]. In contrast, according to Milani et al. [33], the relative abundance of *Actinobacteria* increases with the infant’s age, which was also confirmed in this research since their average representation was the lowest (24.75%) in the faecal samples collected within a week after birth. The abundance of *Verrucomicrobia* in the faeces of the infants increased with the infant’s age, as well as in the microbiota of human milk during lactation.

The results obtained at the bacterial class level also confirmed this connection between the microbiota composition of each mother’s milk and the faeces of their infants, as well as the premise that bacteria from the class *Clostridia* are late colonisers of the child intestines [34]. It has been proven that the relative abundance of *Clostridia* is significantly higher in the faecal samples of children born by caesarean section compared to vaginally born children [35]. The relative abundance of bacteria from the *Actinobacteria* and *Verrucomicrobiae* classes also increased over time in the microbiomes of human milk and infant faeces. A comparison of the average abundance of bacterial classes also confirmed that the microbiota of human milk is more diverse than the intestinal microbiota of infants. Thus, the prevalence of bacteria from 14 taxonomic classes was observed in human milk samples and from only 11 taxonomic classes in infant faecal samples. The obtained results are in accordance with those of Pärnänen et al. [36], who demonstrated that bacteria from the class *Bacilli* predominate in human milk and bacteria from the class *Actinobacteria* predominate in faeces.

Furthermore, *Streptococcus* and *Staphylococcus* were the most abundant genera in the human milk microbiome, which agrees with Zimmermann and Curtis [27], according to which the most abundant bacteria in mother’s milk belong to the genera *Staphylococcus, Streptococcus, Lactobacillus, Pseudomonas, Bifidobacterium, Corynebacterium, Enterococcus, Acinetobacter, Rothia, Cutibacterium, Veillonella* and *Bacteroides*. In the infant faeces samples, the bacteria of the genera *Bifidobacterium* and *Bacteroides* were the most abundant. According to Sela et al. [37], bacteria of the genus *Bifidobacterium* are most abundant in the faeces of infants because they are highly adapted to the breakdown of the complex oligosaccharides present in human milk. The genus *Bacteroides* consists of beneficial bacteria that are highly represented in the neonatal microbiome. They have an important function in the development of the intestinal immune system and, together with bacteria of the genera *Bifidobacterium* and *Lactobacillus*, strengthen the intestinal barrier function by stimulating mucin production and reducing intestinal permeability, which is especially important for the immature neonatal gut [38,39].

Using the metrics Faith’s PD and Observed OTUs, the α-diversity of the human milk microbiome was significantly higher than that of the infant faeces, implying that the human milk microbiota is more diverse than the faecal microbiota, which has also been established by other authors by sequencing 16S rRNA genes [20,21]. While α-diversity estimates the diversity and richness present in a particular sample, it does not show how similar these samples are to each other. The β-diversity represents a comparison of the microbial community of all samples used in the analysis, based on their composition, in order to provide a representation of the similarity of microbial communities. In PCoA analysis, human milk samples are grouped within one cluster, and the infant faecal samples within another. The cluster representing the faecal samples was more compact, implying that the microbiome of infant faeces is more similar in composition than the microbiome of human milk.

The human milk microbiome is recognised as an important source of LAB strains with potential features of next-generation probiotics, including those that produce specific biomolecules with potential therapeutic activity, such as S-layer proteins, EPSs and plantaricins. Indeed, it has been shown that the cell-free supernatant of these bacteria or products containing inactivated bacterial cells, termed postbiotics, mediate the immunomodulatory activity of the host [40]. Therefore, the isolation and characterisation of certain probiotic-produced biomolecules, which may be sufficient to elicit the probiotic response, is a safer alternative for potential clinical application, especially in chronic inflammatory bowel disease, where live probiotic cells have not yet given encouraging results [41].

In order to identify the producers of potential therapeutic biomolecules, a screening of 28 genetically distinct strains selected according to hierarchical cluster analysis of 100 human milk bacterial isolates was performed. Initially, LAB strains with the specific feature of producing S-layer proteins were selected. The results of this study confirmed that even the four *L. brevis* strains isolated from the human milk microbiome produce S-layer proteins. Namely, S-proteins from *Lactobacillus* species have immunomodulatory activity in the gut, while S-proteins from *L. brevis* strains have a regulatory role in modulating the effector functions of dendritic cells (DCs). When the S-layer of *L. brevis* is recognised by the macrophage-inducible C-type lectin (Mincle), this interaction triggers the production of both pro- (IL-6 and TNF) and anti-inflammatory (IL-10 and TGF-β) cytokines, thus creating a balance in the cytokine response [42]. Furthermore, by stimulating the production of IL-6, a cytokine that regulates the production of IL-22 and IL-17 in T-cells, the intestinal immune barrier is strengthened, microbial translocation is limited, and systemic inflammation and its metabolic consequences are prevented. Moreover, S-proteins are of paramount importance as an innovative class of electrochemical biosensors due to their intrinsic ability to self-assemble [43]. No less interesting is the recently proposed application of S-proteins as fusion proteins in multifunctional nanohybrid systems, which are potent and promising candidates for cancer treatment [44].

Regarding the bacteriocinogenic activity of human milk LAB isolates, bacteriocin production is considered an important feature of next-generation probiotics, through which the producing microorganism ensures a competitive advantage in the ecosystem, which is why it represents one of the most important WHO/FAO selection criteria for probiotic strains [45,46]. In addition to antimicrobial activity, plantaricin showed effects on healthy and cancerous epithelial intestinal cell lines by enhancing the viability of healthy cells and reducing the proliferation of cancer cells [47]. The presence of three structural genes (*pln*J, *pln*A and *pln*EF) encoding plantaricins was confirmed by PCR analysis in all *L. plantarum* strains (KR19, MC19, MB7, MB15, MB18 and RS10). *L. plantarum* species have one of the largest genomes among all LAB strains because it comprises a large number of genes obtained by horizontal gene transfer via mobile elements such as prophages, plasmids, transposons and integrons and often contains plasmids that may contain genes for antibiotic resistance, lactose catabolism and the production of proteolytic enzymes and bacteriocins [48]. The bacteriocin *pln* locus contains five operons: *plnEFI* and *plnJKLR*, which encode bacteriocins and immune proteins; *plnGHSTUV*, which encodes an ABC peptide secretion transport system; *plnABCD*, which encodes peptides for the signal transduction pathway; and *plnMNOP*, which contains genes of unknown function. The peptide PlnA induces transcription of all five *pln* operons mentioned above, which are repressed under normal conditions [49]. *L. plantarum* KR19 and MC19 showed the strongest antibacterial activity not only against potentially pathogenic bacterial species but also against related *Lactococcus* and *Enterococcus* strains. Bacteriocins are ribosomally synthesised low molecular weight antimicrobial peptides that primarily act antagonistically against related bacterial species.

EPS is another significant biomolecule specific to a particular bacterial strain. The production of EPS by the strain *Limosilactobacillus fermentum* MC1 was recognised through a specific “ropy” phenotype of grown bacterial colonies. One-dimensional NMR analysis confirmed the production of EPS bound to the cell surface of the MC1 strain. The signals in the anomeric region of the MC1 EPS-b ^1^H NMR spectrum at 5.18 and 5.07 ppm are very similar to those of *L. fermentum* D12 EPS [10], indicating that these two polysaccharides are very similar or even identical, but further structural investigation are needed to confirm these data. Moreover, LAB-produced EPS are frequently used as food additives in dairy foods, gluten-free sourdough bread, plant-based beverages, meat products and functional products (water binding, emulsifier, gelling, stabiliser) [50]. Furthermore, there are some clinical and pharmaceutical applications of EPS, such as intelligent drug delivery systems (micro- and nanosystems for sustained delivery), interpenetrating polymer networks (IPNs), anticancer drug-targeting, recombinant macromolecular biopharmaceuticals, gene therapy, tissue engineering, and the role of EPS in diagnostics [51,52]. As non-digestible polysaccharides, they can also act as prebiotic substrates for the gut microbiota, with prebiotic activity through short-chain fatty acids and lactate production, which are metabolites of EPSs fermentation in the gut and are involved in antibacterial activity, modulation of the immune system, energy supply to intestinal epithelial cells and modulation of cholesterol and lipid metabolism [15].

## 4. Materials and Methods

### 4.1. Collection of Human Milk and Infant Faecal Samples 

Healthy, breastfeeding women with normal body mass index (BMI) and their healthy, vaginally delivered infants who were breastfed for at least one month after solid food was introduced into the infant’s diet were recruited. An exclusion criterion for infants was antibiotic use, whereas the exclusion criterion for women was the presence of metabolic or chronic diseases. The mothers (*n* = 5), voluntary research participants, collected samples of human milk and infant faeces in separate sterile containers at 3 distinct time points: within one week after birth, one month after birth, and one month after the introduction of solid food into the infant’s diet (Table 3). For milk sampling, mothers were asked to clean the breast with water and wash their hands. Samples of human milk were collected into sterile containers by manual expression before breastfeeding, discarding the first few drops of milk. The first subsequent sample of the infant’s faeces was also collected in a separate sterile container. A total of 15 samples of human milk and 15 samples of infant faeces were collected. Permission for collecting and working with breast milk and infant faeces was obtained from the Ethics Committee (No. 380-59-10106-17-100/99, Class 641-01/17-02/01) of the Faculty of Medicine, University of Zagreb, Zagreb, Croatia. The samples were frozen immediately after collection, transported in that state to the Laboratory for Antibiotic, Enzyme, Probiotic and Starter Culture Technologies at the Department of Biochemical Engineering, Faculty of Food Technology and Biotechnology, University of Zagreb, Zagreb, Croatia, and stored at −80 °C until further analysis.

### 4.2. Bacterial Strains

A total of 100 bacterial strains were isolated from human milk samples. *L, helveticus* M92 was used as the S-layer expressing strain (positive control) and *L. plantarum* D13, a non-S-layer producing strain, was used as the negative control in the detection of S-layer proteins [53]. For the assessment of antagonistic activity, the test-microorganisms *Staphylococcus aureus* 3048, *Listeria monocytogenes* ATCC^®^ 19111™, *Escherichia coli* 3014 and *Salmonella enterica* serotype Typhimurium FP1 were used. All strains are deposited in the Culture Collection of the Laboratory for Antibiotic, Enzyme, Probiotic and Starter Culture Technologies, Faculty of Food Technology and Biotechnology, University of Zagreb (CIM-FFTB), Zagreb, Croatia. LAB and test-microorganisms stock cultures are maintained at −80 °C in MRS (BD Difco, Detroit, MI, USA) or BHI (Biolife, Milan, Italy) broth, respectively, supplemented with 15% (*v*/*v*) glycerol (Sigma-Aldrich, Saint Louis, MO, USA).

### 4.3. Bacterial Isolation, DNA Extraction and RAPD-PCR-Based Fingerprinting

A total of 100 bacterial isolates, 20 from each mother (*n* = 5), were pooled by randomly selecting colonies grown on MRS agar plates at 37 °C corresponding to the highest dilution at which growth occurred. Each selected colony was subcultured to purity on MRS agar plates and stored in CIM-FFTB, as described.

Genomic DNA of the 100 selected isolates was extracted using the Wizard^®^ Genomic DNA Purification Kit (Promega, Madison, WI, USA) according to the manufacturer’s recommendations. DNA concentration and purity were measured using a BioSpec-Nano spectrophotometer (Shimadzu, Kyoto, Japan), and the isolated DNA was stored at −20 °C. The RAPD-PCR reaction mixtures contained 12.5 µL EmeraldAmp MAX HS PCR Master Mix 2× Premix (TaKaRa, Ohtsu, Japan), 0.5 µL universal M13 (5′-GAGGGTGGCGGTTCT-3′) primer [54], 2 µL of DNA template and EmeraldAmp dH_2_O to adjust the mixture volume to 25 µL. The reaction was carried out in an ABl 2720 PCR device (Applied Biosystems, Foster City, CA, USA) with an initial denaturation step at 94 °C for 1 min, 35 cycles of denaturation at 94 °C for 1 min, annealing at 40 °C for 20 s and extension for 80 s at 72 °C, followed by 5 min of final extension at 72 °C. The amplified PCR products were electrophoretically separated on the agarose (Invitrogen, Waltham, MA, USA) gel (1% *w*/*v*) at 60 V, stained with ethidium bromide (Boehringer Mannheim GmbH, Baden-Wurttemberg, Germany) (0.5 µg/mL) and visualised with ultraviolet light on a MiniBIS Pro transilluminator at a wavelength of 254 nm using the Gel Capture program (DNR Bio-Imaging Systems Ltd., Neve Yamin, Israel) version 7.1. The λ DNA *Hind*III (Fermentas, Waltham, MA, Canada) and the 100 bp DNA Ladder (Invitrogen, Waltham, MA, USA) were used as molecular size standards. 

In order to exclude genetically identical isolates, a hierarchical cluster analysis was performed by comparing the RAPD-PCR electrophoretic profiles of 100 human milk isolates using GelCompar II software (Applied Maths, Sint-Martens-Latem, Belgium). The results are disclosed in the form of a dendrogram. Finally, one representative of each cluster generated by the dendrogram, resulting in total of 28 bacterial isolates, was selected for further analysis.

### 4.4. LAB Selection and 16S rRNA Identification

The selected 28 isolates were further characterised based on their cell morphology, Gram-reaction, KOH method, sporulation, and catalase and API 50 CHL assays (BioMérieux, Marcy-l’Étoile, France) [55]. Genotypic identification was performed by sequencing the 16S rRNA gene. The reaction mixture for the amplification step of the 16S rRNA region contained 1× GoTaq Flexi buffer without MgCl_2_ (Applied Biosystems, Foster City, CA, USA), 1.5 mM MgCl_2_ (Applied Biosystems, Foster City, CA, USA), 0.025 U/µL Taq polymerase (TaKaRa, Ohtsu, Japan), 1.5 µM primers UNI16SF (5′-GAGAGTTTGATCCTGGC-3′) and UNI16SR (5′-AGG AGG TGA TCC AGC CG-3′) [56], 0.2 mM deoxynucleoside triphosphates (dNTPs) (TaKaRa, Ohtsu, Japan) and 1 ng/µL DNA. The PCR programme consisted of an initial denaturation at 96 °C for 5 min, 30 cycles of 30 s at 96 °C, 30 s at 55 °C and 30 s at 72 °C, followed by 5 min of final elongation at 72 °C. The resulting PCR products were purified using the QIAquick PCR Purification Kit (QIAGEN, Hilden, Germany), according to the manufacturer’s recommendations and sequenced at Macrogen (Amsterdam, The Netherlands). The 16S rRNA sequences were aligned with the NCBI nt database, using the BLASTn algorithm v. 2.11.0 [57] to find nucleotide sequence homologues (https://blast.ncbi.nlm.nih.gov/Blast.cgi, accessed on 18 February 2020).

### 4.5. WGS Identification of LAB Producers and Detection of Potential Therapeutic Biomolecules 

#### 4.5.1. Detection of S-Layer Proteins by SDS-PAGE and LC/MS Methods

A slightly modified method by Uroić et al. [58] was used to extract S-layer proteins from the cell surfaces of selected LAB cells isolated from human milk. *Lactobacillus* cells were grown in MRS broth to an OD_620_ nm of 2.0, washed and treated with Laemmli buffer. Samples were boiled and subjected to Dodecyl Sulphate Polyacrylamide Gel Electrophoresis (SDS-PAGE), as described by Kos et al. [59]. Electrophoretic separation of proteins was performed at a constant voltage of 100 V, along with the ProSieve QuadColor Protein Marker 4.6–315 kDa (Lonza, Morristown, NJ, USA). Gel images were characterised using the HP Scanjet 3800 Photo Scanner, and the obtained electrophoretic profiles were analysed using the GelCompar II software (Applied Maths, Sint-Martens-Latem, Belgium) and depicted in the form of a dendrogram. Identification of the extracted S-layer proteins was achieved, according to Banić et al. [53]. Briefly, protein bands corresponding to a molecular size in the range of 45–55 kDa were excised from the SDS-PAGE gel, trypsinised and subjected to an LC-MS combined with a Mascot database search. 

#### 4.5.2. Detection of Plantaricins by PCR and Agar Spot-Test 

Potential plantaricin producers isolated from human milk were selected based on their genotypic and phenotypic characterisation antagonistic activity, according to Butorac et al. [9]. PCR screening of plantaricin structural genes was performed using six primers, *pln*A, *pln*EF, *pln*J, *pln*NC8, *pln*S and *pln*W, under the described conditions. Antimicrobial activity was assessed against four different test microorganisms, *S. aureus* 3048, *L. monocytogenes* ATCC^®^ 19111™, *E. coli* 3014 and *S.* Typhimurium FP1 by agar spot-test method [23]. 

#### 4.5.3. Detection of Exopolysaccharides by ^1^H NMR Method

The isolation of EPSs of *L. fermentum* MC1, after “ropy” phenotype detection, was performed by alkaline treatment of the bacterial cells according to Ferrari et al. [60] with slight modifications. In brief, strain *L. fermentum* MC1 was cultivated in 500 mL of MRS broth (BD Difco, Detroit, MI, USA) supplemented with 2% glucose (Kemika, Zagreb, Croatia). Cells were harvested by centrifugation (4000× *g*, 30 min, 4 °C), washed with sterile 0.9% NaCl solution and treated overnight with 1 vol of 2 M NaOH under a magnetic stirrer. EPSs released from the cell surface were extracted from the supernatant by centrifugation (8000× *g*, 30 min, 4 °C) and treated with four volumes of ethanol (Kemika, Zagreb, Croatia) followed by overnight incubation at −20 °C. The pellets obtained by centrifugation (8000× *g*, 30 min, 4 °C) were dissolved in distilled water and dialysed against distilled water, using 10–14 kDa MWCO dialysis tubes (Spectrum Laboratories, Los Angeles County, CA, USA). Finally, the purified EPSs-b were recovered by freeze-drying (Martin Christ Gefriertrocknungsanlagen GmbH, Osterode am Harz, Germany). For ^1^H NMR spectroscopy experiments, the polysaccharide (10 mg) was exchanged twice with 99.9% D_2_O (Sigma-Aldrich, Saint Louis, MO, USA) by lyophilisation, dissolved in 0.6 mL of 99.96% D_2_O and introduced into a 5 mm NMR tube for data acquisition. Spectra were recorded using a 500 MHz VARIAN UNITY INOVA NMR spectrometer (Kenilworth, NJ, USA) operating at 50 °C. Chemical shifts are expressed in ppm using acetone as an internal reference (2.225 ppm for ^1^H). The NMR spectra were processed with the MestreNova software.

#### 4.5.4. Whole Genome Sequencing (WGS)

The genomic DNA of selected LAB strains, potential producers of therapeutic biomolecules, was extracted using a Maxwell^®^ DNA Cell kit in a Maxwell^®^ 16 Research System instrument (Promega, Madison, WI, USA). WGSwas conducted on an Illumina MiSeq 2500 platform (Illumina, San Diego, CA, USA) at IGA Technology Services (IGA Technology Services Srl, Udine, Italy) using a paired-end approach [53]. This Whole Genome Shotgun project was submitted to the NCBI nt database (BioProject PRJNA388578, version 1.0) under the accession numbers listed in the Table 4 for each strain with the following feature: biosynthesis of S-layer proteins, plantaricin and EPSs [22]. 

### 4.6. Microbiome Analysis with QIIME 2™—Determination of Taxonomy and α- and β-Diversity

Total genomic DNA from human milk (5 mL) and infant faecal (100 mg) samples were extracted with Maxwell DNA Tissue Kit using an automated extraction platform, the Maxwell^®^ 16 Research System instrument (Promega, Madison, WI, USA). Prior to the DNA isolation, bacterial cells were lysed for 2 h in 200 µL of lysozyme (EuroBio, Les Ulis, France) solution (5 mg/mL) in TE [Tris-EDTA (Ethylenediaminetetraacetic acid)] buffer and sonicated with Sonopuls mini20 (Bandelin electronic GmbH & Co. KG, Berlin, Germany). Amplification of the V1-V3 hypervariable regions of the 16S rRNA gene was performed at Molecular Research LP (MRDNA, Shallowater, TX, USA) on the Illumina MiSeq platform using ill27Fmod (5′-AGRGTTTGATCMTGGCTCAG-3′) and ill519Rmod (5′-GTNTTACNGCGGCKGCTG-3′) primers following the manufacturer’s instructions at MRDNA. Raw 1danaata were obtained as FASTQ files and analysed using the QIIME 2™ v. 2021.4 [61] bioinformatics pipeline with default parameters [62]. Each sequence is demultiplexed using the q2-demux plugin based on a defined nucleotide sequence, unique to each sample, located at the beginning of each read sequence (barcode) and quality filtered followed by sequence joining, denoising and creating a table of Amplicon Sequence Variants (ASV) with q2-DADA2 plugin. Obtained ASVs were aligned using the mafft algorithm and used to construct a phylogenetic tree using the Fasttree tool via the q2-phylogeny plugin. The obtained data were utilised to estimate the alpha (α) and beta (β) diversity of the samples, for which it was necessary to determine the sequencing depth from the DADA2 feature Table. A- diversity metrics (“Observed OTUs”, “Shannon” and “Faith’s Phylogenetic Diversity (PD)”), beta diversity metrics (unweighted UniFrac), and Principle Coordinate Analysis (PcoA) were estimated using q2-diversity after samples were rarefied to 900 sequences per sample, assigned as rarefaction depth. Samples containing a lower number of required sequences were omitted from the downstream diversity analyses. Taxonomy was assigned to each ASV using the q2-feature-classifier and the classify-sklearn naïve Bayes classifier against the Greengenes 13_8 99% OTUs reference sequences. In order to determine the differences in the representation of individual ASVs between samples or groups of samples, the ANCOM method was used. The comparison of α-diversity indices of human milk and infant faecal samples was performed using the Two-Way ANOVA.

### 4.7. Statistical Analysis

Based on the Observed OTUs, Faith’s PD, and Shannon indices, differences in bacterial diversity of the human milk microbiome and the faecal microbiome of infants were assessed by two-way analysis of variance (ANOVA) with Geisser–Greenhouse correction followed by the post hoc Tukeyʹs multiple comparisons test. Analysis was performed using GraphPad Prism v9.4.1 (GraphPad Software, San Diego, CA, USA).

## 5. Conclusions

The influence of the composition of the microbiome of human milk on the microbiome of the faeces of infants was observed at the level of species, class and genus. The amplicon sequencing data analysis revealed that each human milk and infant’s faeces microbiome is unique. The composition of the human milk microbiome changes during lactation, as does the infant faecal microbiome. The α-diversity of the analysed microbiomes revealed that the human milk microbiota is more diverse, i.e., species-rich with a high portion of *Lactobacillus* species observed, compared to the infant faecal microbiota. Out of 100 strains isolated from human milk, 28 genetically distinct isolates were obtained by hierarchical cluster analysis of RAPD-PCR electrophoretic profiles. Potential next-generation probiotic strains isolated from human milk were further selected based on features for the synthesis of potential therapeutic biomolecules, the production of which is strain-dependent and rather rare. Four *L. brevis* strains express S-layer proteins, while six *L. plantarum* strains produce plantaricins, among which two strains have shown the strongest antibacterial activities against potentially food-spoilage and pathogenic bacteria. Only *L. fermentum* MC1 out of the hundred human milk isolates synthesised EPSs. These findings also encourage research aimed at characterising the role of human milk isolated *Lactobacillus* in host-intestinal microbiota interactions of infants. All of the analysed strains and their potential therapeutic biomolecules will be studied as potential next-generation probiotics in the future. 

## Figures and Tables

**Table 1:** 16S rRNA gene sequence identification of selected bacterial human milk isolates.

Strain	Identification	% identity	Accession Number	e-Value
KR19	*Lactiplantibacillus plantarum*	98	MG550988.1	0.0
KR20	*Enterococcus faecium*	98	MN566092.1	0.0
MC1	*Limosilactobacillus fermentum*	98	KY435712.1	0.0
MC2	*Enterococcus faecalis*	98	KC113205.1	0.0
MC5	*Staphylococcus epidermis*	98	CP035643.1	0.0
MC13	*Enterococcus faecium*	97	CP014529.1	0.0
MC19	*Lactiplantibacillus plantarum*	97	HM130542.1	0.0
AF2	*Staphylococcus epidermidis*	96	AY030340.1	0.0
AF4	*Staphylococcus epidermidis*	97	CP014119.1	0.0
AF5	*Staphylococcus epidermis*	98	CP043804.1	0.0
AF12	*Enterococcus durans*	97	MF405179.1	0.0
AF16	*Enterococcus faecium*	98	JN560856.1	0.0
MB1	*Levilactobacillus brevis*	85	MK774569.1	0.0
MB2	*Levilactobacillus brevis*	84	CP031208.1	3 × 10^−7^
MB5	*Streptococcus oralis* subsp. *dentisani*	83	CP034442.1	0.0
MB6	*Staphylococcus epidermis*	98	CP043804.1	0.0
MB7	*Lactiplantibacillus plantarum*	95	MF197402.1	0.0
MB10	*Streptococcus salivarius*	97	CP014144.1	0.0
MB11	*Streptococcus oralis*	98	LR134336.1	0.0
MB13	*Levilactobacillus brevis*	88	MT512175.1	0.0
MB15	*Lactiplantibacillus plantarum*	97	JQ801725.1	0.0
MB18	*Lactiplantibacillus plantarum*	90	MT604681.1	0.0
MB20	*Levilactobacillus brevis*	88	JQ805655.1	0.0
RS4	*Streptococcus oralis*	92	CP019562.1	0.0
RS8	*Staphylococcus epidermis*	98	AP019721.1	0.0
RS10	*Lactiplantibacillus plantarum*	98	AB362728.1	0.0
RS17	*Staphylococcus epidermidis*	89	MG557813.1	0.0
RS19	*Streptococcus mitis*	98	KX880968.1	0.0

**Table 2:** S-layer proteins of *L. brevis* MB1, MB2, MB13 and MB20 identified by SDS-PAGE coupled with LC-MS and Mascot database search.

Peptide Monoisotopic Neutral Mass	Protein Score	MS/MS Score	Significance	Peptide Sequence
*L. brevis* **MB1**				
1271.7085	484	45	no	K.VNIVDLSGNTIK.S
1466.7405		98	unique	K.AFGPDFAAAITSATK.G
1635.7781		100	yes	K.AFGPDFAAAITSATK.G
1694.8363		103	unique	K.DALVAAGVLYDSTSDAK.A
2499.2969		138	yes	K.ADGWILLSNLTQTNALNEATQVK.V
*L. brevis* **MB2**				
1271.7085	462	36	no	K.VNIVDLSGNTIK.S
1466.7405		87	unique	K.AFGPDFAAAITSATK.G
1635.7781		106	yes	K.AFGPDFAAAITSATK.G
1694.8363		92	unique	K.DALVAAGVLYDSTSDAK.A
2499.2969		140	yes	K.ADGWILLSNLTQTNALNEATQVK.V
*L. brevis* **MB13**				
1271.7085	437	75	unique	K.VNIVDLSGNTIK.S
1466.7405		77	unique	K.AFGPDFAAAITSATK.G
1635.7781		109	unique	K.SATAFAGGLTSYDTFK.E
1694.8363		108	unique	K.DALVAAGVLYDSTSDAK.A
2499.2969		118	unique	K.ADGWILLSNLTQTNALNEATQVK.V
*L. brevis* **MB20**				
1466.7405	647	85	unique	K.AFGPDFAAAITSATK.G
1635.7781		107	unique	K.SATAFAGGLTSYDTFK.E
1694.8363		103	unique	K.DALVAAGVLYDSTSDAK.A
1890.0211		21	no	R.NVNLTGTNAIYTKPGTVK.G
2259.1383		21	no	K.TIADTTAYKDATFSVDKVGTR.T
2499.2969		141	unique	K.ADGWILLSNLTQTNALNEATQVK.V

**Table 3:** Labels of human milk and infant faecal samples used in this paper.

Nursing Mother	Sampling Time	Human Milk	Infant’s Faeces
**KR**	within one week after birth	KRM1	KRF1
	one month after birth	KRM2	KRF2
	one month after the introduction of solid food into the infant’s diet	KRM3	KRF3
**MC**	within one week after birth	MCM1	MCF1
	one month after birth	MCM2	MCF2
	one month after the introduction of solid food into the infant’s diet	MCM3	MCF3
**AF**	within one week after birth	AFM1	AFF1
	one month after birth	AFM2	AFF2
	one month after the introduction of solid food into the infant’s diet	AFM3	AFF3
**MB**	within one week after birth	MBM1	MBF1
	one month after birth	MBM2	MBF2
	one month after the introduction of solid food into the infant’s diet	MBM3	MBF3
**RS**	within one week after birth	RSM1	RSF1
	one month after birth	RSM2	RSF2
	one month after the introduction of solid food into the infant’s diet	RSM3	RSF3

**Table 4:** Accession numbers of identified LAB strains, potential producers of the therapeutic biomolecules.

Strain	Identification	Accession Number	Biomolecule
**MB1**	*Levilactobacillus brevis*	SAMN22155538	S-layer protein
**MB2**	*Levilactobacillus brevis*	SAMN22155539	S-layer protein
**MB13**	*Levilactobacillus brevis*	SAMN22155540	S-layer protein
**MB20**	*Levilactobacillus brevis*	SAMN22155541	S-layer protein
**MC19**	*Lactiplantibacillus plantarum*	SAMN22155542	Plantaricin
**KR19**	*Lactiplantibacillus plantarum*	SAMN22155536	Plantaricin
**MC1**	*Limosilactobacillus fermentum*	SAMN22155537	EPS

## Data Availability

Not applicable.

## Supplementary Materials

The supporting information can be downloaded at: https://www.mdpi.com/article/10.3390/ijms232214382/s1.
