# Supplementary figures and images for: The Human Milk Microbiota Produces Potential Therapeutic Biomolecules and Shapes the Intestinal Microbiota of Infants
Source: Int J Mol Sci. 2022 Nov 19;23(22):14382. doi: 10.3390/ijms232214382 (PMC9699365; doi:10.3390/ijms232214382)

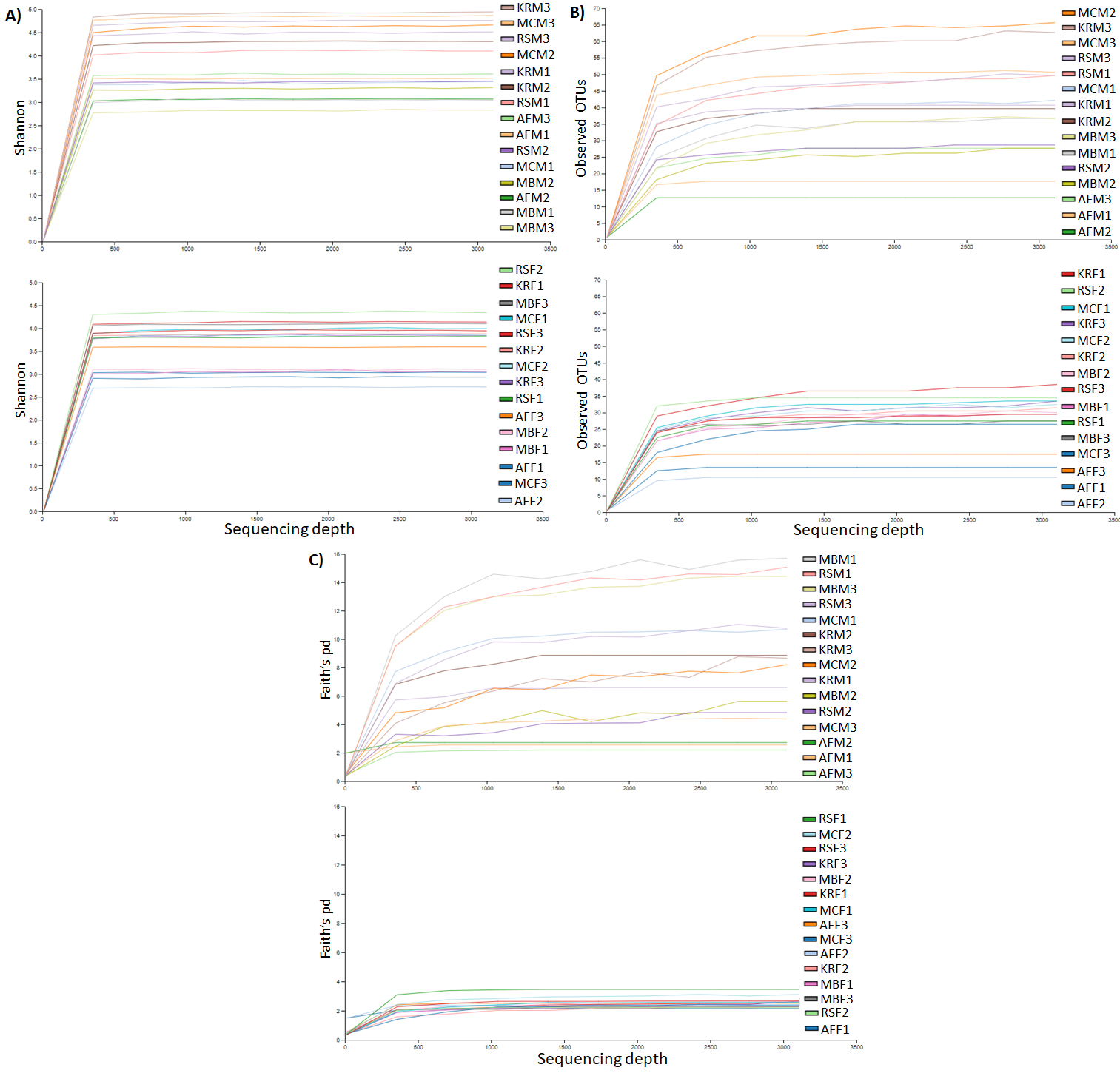

Supplement: Supplementary file 1 [file ijms-23-14382-s001.zip › Supplementary figure S1.tif]

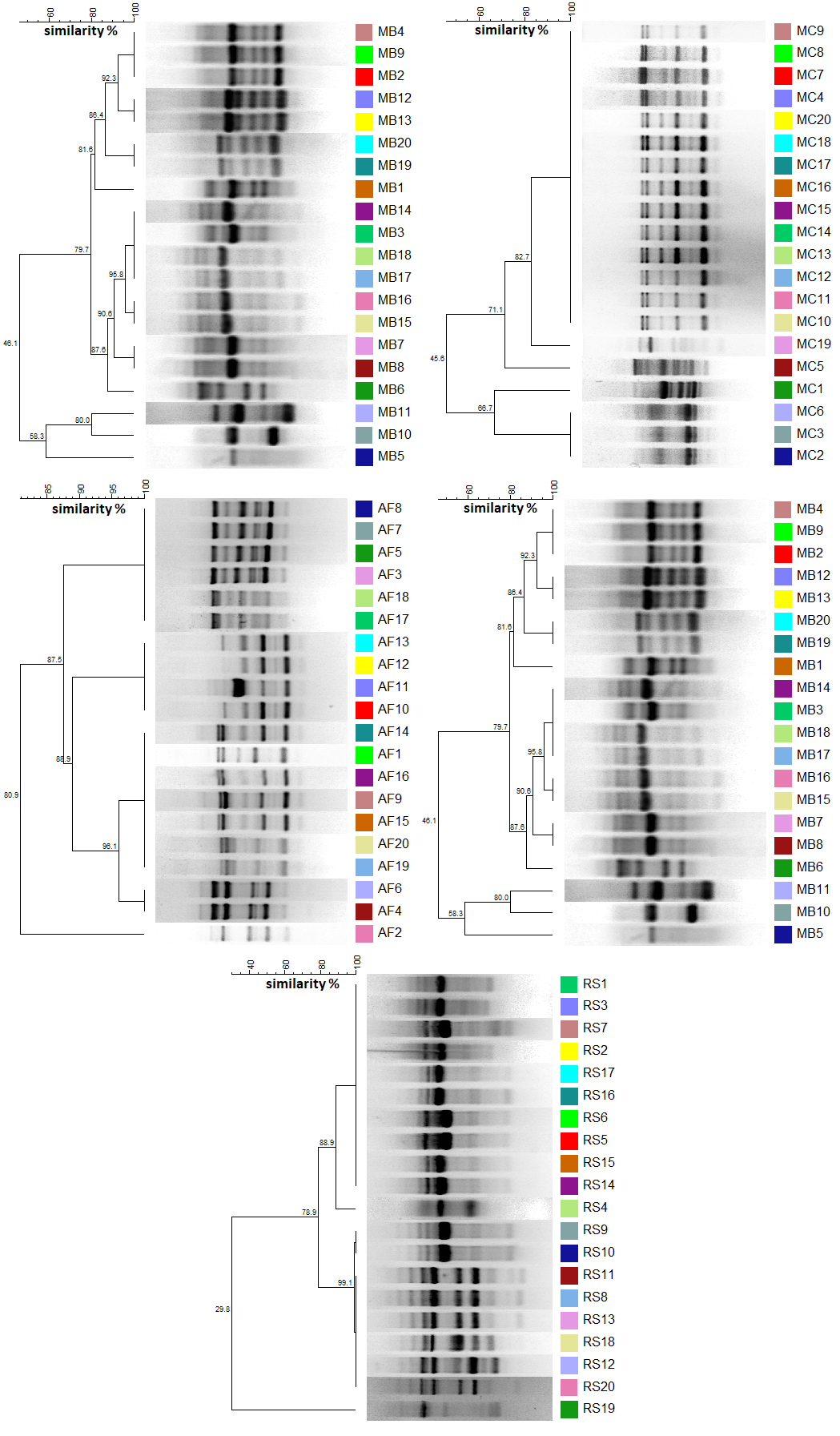

Supplement: Supplementary file 1 [file ijms-23-14382-s001.zip › Supplementary figure S2.tif]

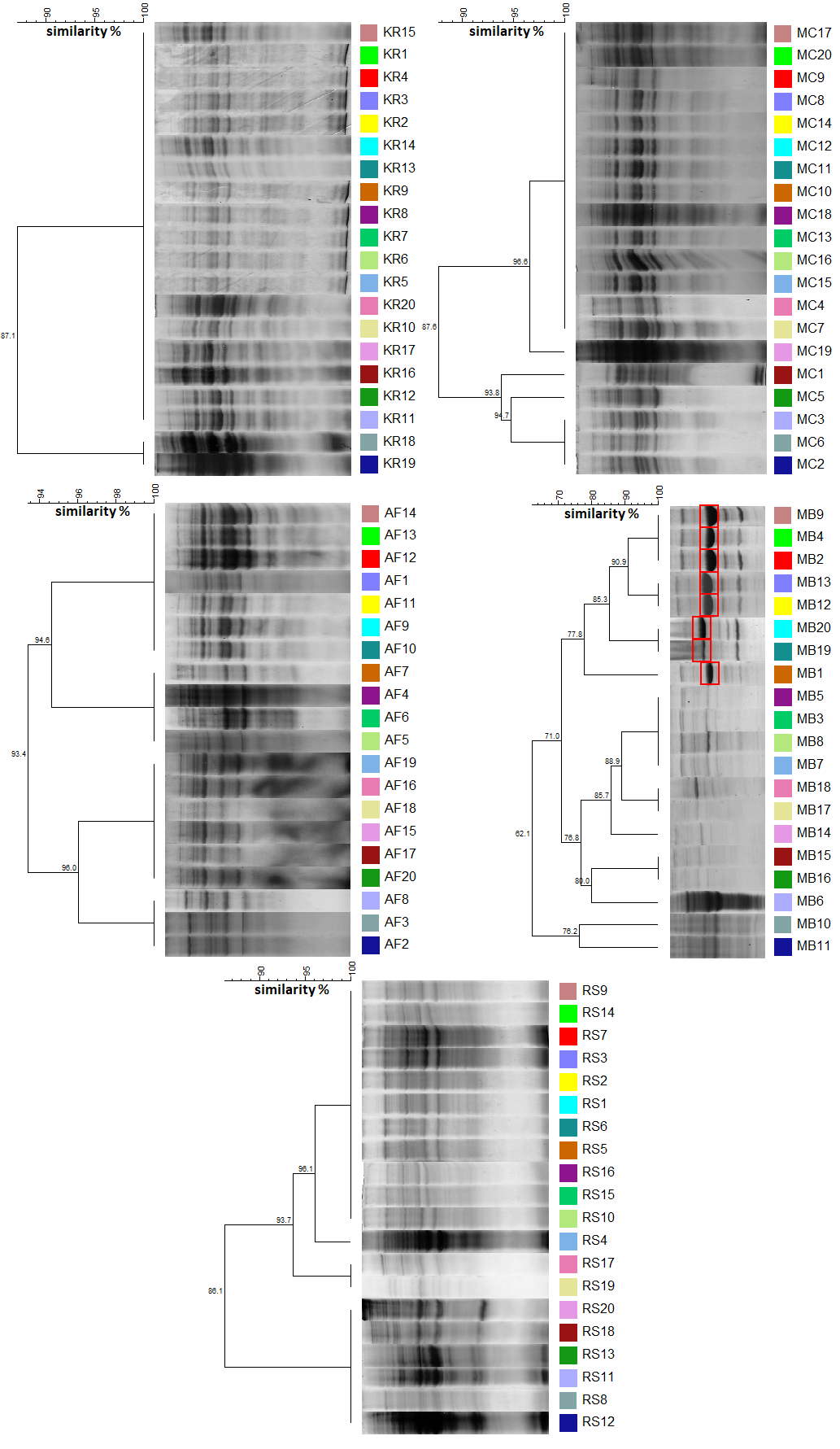

Supplement: Supplementary file 1 [file ijms-23-14382-s001.zip › Supplementary figure S3.tif]

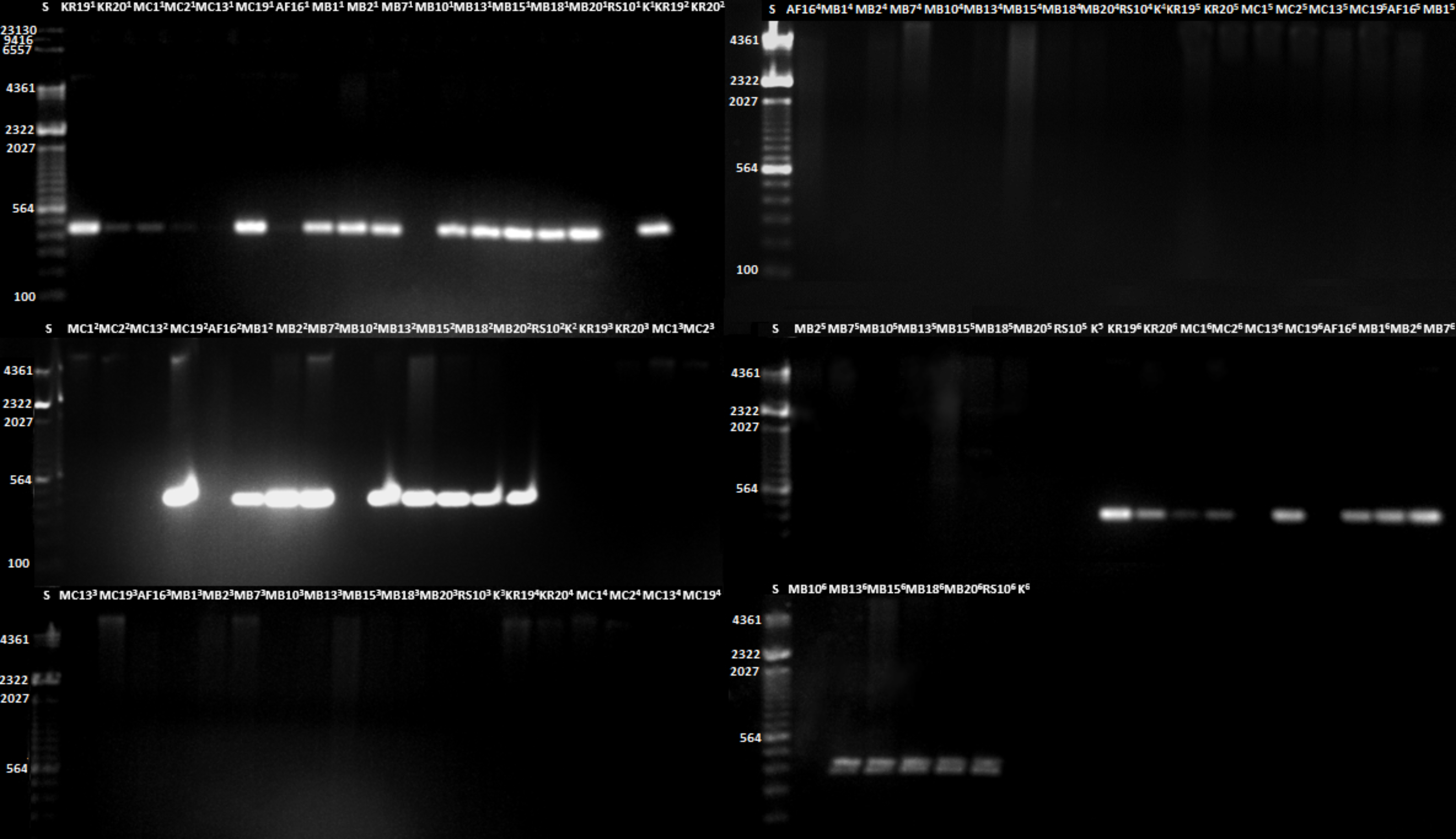

Supplement: Supplementary file 1 [file ijms-23-14382-s001.zip › Supplementary figure S4.tif]
